# Comparison of three formulae for estimation of glomerular filtration rate in severely malnourished children at tertiary care facility

**DOI:** 10.12669/pjms.336.13675

**Published:** 2017

**Authors:** Misbah Anjum, Khemchand N. Moorani, Bilquis Naeem, Shazia Kulsoom, Ashfaq Ahmed Memon

**Affiliations:** 1 Misbah Anjum, MBBS, FCPS. Assistant Professor, Dept. of Pediatric Medicine (Unit-III), National Institute of Child Health (NICH), Jinnah Sindh Medical University (JSMU), Karachi, Pakistan; 2 Prof. Khemchand N. Moorani, FCPS, MCPS, MBBS, Pediatric Nephrology & Medical (Unit-III), National Institute of Child Health (NICH), Jinnah Sindh Medical University (JSMU), Karachi, Pakistan; 3 Bilquis Naeem, MBBS, FCPS. Senior Registrar, Pediatric Nephrology & Medical (Unit-III), National Institute of Child Health (NICH), Jinnah Sindh Medical University (JSMU), Karachi, Pakistan; 4 Shazia Kulsoom, MBBS, FCPS. Senior Registrar, Dept. of Pediatric Medicine (Unit-III), National Institute of Child Health (NICH), Jinnah Sindh Medical University (JSMU), Karachi, Pakistan; 5 Ashfaq Ahmed Memon, M. Sc Statistics. Senior Statistical Officer, Pakistan Health Research Centre (PHRC), Specialized Research Centre on Child Health, National Institute of Child Health (NICH), Jinnah Sindh Medical University (JSMU), Karachi, Pakistan

**Keywords:** eGFR formula, Glomerular filtration rate, Renal function impairment, Severe acute malnutrition

## Abstract

**Objectives:**

First objective was to compare eGFR by Updated Schwartz (US) and Simple Height Independent (SHID) formula with Original Schwartz (OS) in children with Severe Acute Malnutrition (SAM). The second objective was to compare eGFR in children below and above two years.

**Methods:**

This analytic study on estimation of GFR was based on retrospective data collected from 78 children with SAM at Nutritional Rehabilitation Unit from October 2014 - March 2015. Glomerular filtration rate was calculated using serum creatinine (S. Cr) and height in Original Schwartz, US and by age in SHID equation and compared with OS as standard. Data was analyzed using descriptive statistics.

**Results:**

There were 78 children in this study. Males were 39(50%). Mean age of patients was 18±15.53 months with 62(79.48%) ≤24 months. Mean weight, height and Mid Upper Arm Cir-cumference was 5.69±2.42kg, 68.52+13.59 cm and 10±1.57 cm respectively. Mean eGFR by OS, US and SHID formula was 71.45±49.89, 58.06±3.91 and 59.33±3.73ml/min/1.73m2 respectively. There was significant difference (0.001) in mean eGFR calculated by three different formulae. Majority of children (73%) had subnormal GFR (<90 ml/min /1.73 m2). There was a significant difference in GFR ≥90ml calculated by US compared to OS (0.025) and by SHID with OS (0.04) in children below two years and no difference in children above two years. But there was no difference in other categories of eGFR calculated by either of formula in both age groups.

**Conclusion:**

We found a significant difference in eGFR in ranges above 90 ml/min/1.73 m2 by US compared to OS as well as by SHID with OS in children below two years and no difference in children above two years. Also, there was no difference in GFR categories below 90 ml/min /1.73 m2 calculated by either of formula in both age groups. So, we may conclude that either of formula can be used in clinical practice for eGFR in mild to severe renal dysfunction in severely malnour-ished children.

## INTRODUCTION

Glomerular filtration rate (GFR) is most important measure of kidney function. The accurate measurement of GFR is important in clinical practice in establishing diagnosis of acute and chronic renal damage, for adjustment of drug doses, in intensive care settings as well as in outpatient follow up and in organ transplantation.^1^-^3^

Many methods for assessment of accurate GFR are available but require administration of substances like inulin,^51^Cr-EDTA, iohexol or ^99m^TC-diethylene triamine penta- acetic acid.^4^,^5^ Gold standard for measurement of GFR is renal clearance of inulin but this is not commonly used due to its relative invasiveness and time consuming.^1^ Other methods use measurement of endogenous clearance of creatinine (Ccr) or the clearance of filtration markers like ^51^Cr-EDTA or iothalamate.^1^ These drugs are freely filtered from glomerulus and are neither secreted nor absorbed by tubules.^5^ These methods require either accurate and timed urine collection or injection of radio-labelled compounds with multiple sampling of blood.^1^ But measuring GFR from these techniques is difficult to perform, time consuming, invasive and are not available in all health care facilities.^1^,^5^

To avoid these problems alternate bedside formulas have been developed to estimate GFR using serum creatinine (S. Cr) as a marker of renal function.^1^,^2^,^5^ For pediatric population most frequently used is Schwartz formula.^1^ Other formulas are updated Schwartz(US), Simple height independent(SHID), FM equation, Q age and Q height equation.^1^,^5^

Many factors affect the level of S. Cr including variability of muscle mass, gender, age, diet, amount of Cr filtered by glomerulus, tubular secretion and tubular reabsorption.^1^,^6^,^7^ This renders a problem in calculating GFR in children. At birth GFR is low, at 24 hours S. Cr falls and GFR rises progressively and continues to rise till the age of 18 months when it is normalized with body surface area and become independent of age.^7^

Updated Schwartz is derived from the children with GFR range of 15-75 ml/min/1.73 m^2^ using Iohexol clearance, so its application to children with mild renal insufficiency is also questionable.^5^ SHID equation is adjusted for age so it can be used in children. So, its clinical application may be useful when height is not available.^5^

Though, validation of eGFR in pediatric population has been carried out from India and in adults with chronic kidney disease (CKD) from Pakistan.^8^,^9^ Yet there is no data available about eGFR formula in our children with CKD and severe acute malnutrition (SAM). Keeping in view, this specialized group of children, we analyzed the eGFR based on serum creatinine with the aim to provide local data on use of different formula in children with SAM. The main objective of our study was to compare GFR estimated by Updated Schwartz (US) and Simple Height Independent (SHID) formulas with Original Schwartz (OS) in children with SAM. The second objective was to compare eGFR in children below and above 2 years.

## METHODS

This analytic study was carried out on retrospective data collected from 78 children with SAM in the National rehabilitation unit (NRU) at National Institute of Child Health (NICH) Karachi from October 2014 - March 2015. Ethical approval was taken from hospital review committee. Children aged 2-60 months with SAM (either bilateral pitting edema or weight for height z-score -3), admitted in NRU during the study period were enrolled. Formal consent from parents or care givers was taken and children were excluded if refused or left against medical advice.

The clinical information including age, gender, presenting illness, physical findings like weight, height, edema, mid upper arm circumference (MUAC) and laboratory data including complete blood count, serum electrolytes, calcium and phosphorus was obtained from record. Serum creatinine (S Cr) was measured by Jaffe reaction and applied in GFR estimation in three formulas as follows.^10^ *Original Schwartz (OS):* eGFR = k x height (cm) /S Cr (mg) where k = 0.45 for full term infants, 0.33 for preterm infants, 0.55 for children above 12 months. *Updated Schwartz (US):* eGFR = k x height (cm) / S Cr (mg) where k = 0.413 and Simple height independent (SHID) equation: eGFR = 107.3 / S Cr /Q where Q = 0.0270 x age in years +0.2329. Original Schwartz formula was taken as standard and US and SHID formulas were compared with OS using student t-test.

Estimated glomerular rate was categorized according to severity of renal impairment as recommended by Kidney Diseases Initiative Global Outcome (KDIGO) guidelines. According to these guidelines severity is categorized as ≥ 90 ml/min/1.73 m^2^, 60-89 ml, 30-59 ml, 15-29 ml and < 15 ml/min /1.73 m^2^.^11^ GFR calculated by US and SHID equation was compared with OS in each GFR category. We also compared the severity of eGFR calculated by above formulae in two age groups; ≤2 Years and >2 Years.

Data was entered and analyzed using SPSS version 16. For categorical variables, frequency and for numerical variables mean ±SD was calculated. Estimated GFR by US and SHID equation was compared with OS using student t-test. P-value < 0.05 was considered as significant.

## RESULTS

There were 78 children in this study. Males were 39(50%). Mean age of patients was 18±15.53 months. 62(79.48%) of children were ≤2 years of age while 16(20.51%) were above two years.

Demographic, clinical and biochemical features in the study population are shown in Table-I. Mean weight, height and mid upper arm circumference (MUAC) was 5.69±2.42 kg, 68.52±13.59cm and 10±1.57cm respectively. Most (82.05%) of children were marasmus whereas, 17.9% had edematous malnutrition.

**Table-I:** Demographic, clinical and laboratory features of children with severe acute malnutrition.

*Demography & Anthropometry*					
Gender	Female 39 (50%)					
	Male 39 (50%)					

Age groups	*Age (months)*		*Weight(kg)*	*Height(cm)*	*MUAC(cm)
	*N (%)*	*mean ± SD*	*mean ± SD*	*mean ± SD*	*mean ± SD*

Overall	78(100)	18±15.53	5.69±2.42	68.52±13.59	10±1.57
≤ 2 years	62(79.49)	11.35±7.1	6.07±9.98	63.67±10.13	9.781.57
> 2 years	16(20.51)	44.25±10.7	8.58±1.7	87.33±6.73	10.95±1.19

*Clinical Presentations*					*N (%)*	

Diarrhea					41 (52.6)	
Pneumonia					28 (35.9)	
Dehydration					19 (24.4)	
Persistent Diarrhea					17 (21.8)	
Edema					14 (17.9)	
Sepsis					14 (17.9)	
Hypothermia					7 (8.9)	
Others					8 (10.3)	

*Laboratory Parameters*					*(mean ± SD)*	

Hemoglobin (G/dl)					8.66±2.4	
Urea (mg/dl)					29.02±17.37	
Creatinine (mg/dl)					0.68±0.48	
Na (mEq/L)					137.05±6.715	
K (mEq/L)					3.97±1.16	
HCO_-3_ (mEq/L)					21.57±7.4	

* MUAC: mid upper arm circumference

Common reasons for admission of children with SAM (Table-I) were acute watery diarrhea 41(52.6%), anemia 34(43.6%), pneumonia 28(35.9%), persistent diarrhea 17(21.8%), septicemia 14(17.9%) and others 8(10.2%). Fourteen (24.4%) children were dehydrated and 7(8.9%) were hypothermic at the time of admission. Laboratory parameters (Table-I) shows that mean Hb, urea, Cr, Na^+^, K^+^, and HCO_3_^-^ were 8.66±2.4 g/dl, 29.02±17.37 mg/dl, 0.68±0.48 mg/dl, 137.05+6.71 mEq/L, 3.97±1.16 mEq/L, 21.57±7.4 mEq/L respectively.

By Schwartz formula, mean eGFR was 71.45±49.89 ml/min /1.73 m^2^ (9.54-261 ml). Majority of children (73%) had subnormal eGFR (< 90 ml/min /1.73 m2). Comparative estimated GFR using different formulas based on severity of renal impairment is shown in Table-II. This table shows that mean eGFR by OS, US and SHID formula was 71.45±49.89, 58.06±3.91 and 59.33±3.73ml/min/1.73m^2^ respectively. There was significant difference (0.001) in mean eGFR calculated by three different formulae.

**Table-II:** Comparative eGFR Categories using different formulas based on Severity of Renal Impairment in Children with Severe Acute Malnutrition (N=78).

	OS*	US**	SHID***	*US vs OS*	*SHID vs OS*
eGFR ml /min/1.73 m^2^ mean ±SD	71.45±49.89	58.06±3.91	59.33±3.73	0.001	0.001
≥90ml (N, %)	21(26.92)	9(11.54)	10(12.82)	0.025	0.04
60-89 ml	18(23.08)	22(28.21)	23(29.49)	0.58	0.46
30-59 ml	27(34.62)	33(42.31)	33(42.31)	0.41	0.41
15-29 ml	8(10.26)	9(11.54)	9(11.54)	1.00	1.00
<15 ml	4(5.13)	5(6.41)	3(3.85)	0.500	1.00

* OS: Original Schwartz formula,

** US: updated Schwartz,

*** SHID: Simple height independent

On comparison of US with OS, there was a significant difference in GFR ≥90ml (p-value 0.025) whereas there was no difference in other GFR categories below 90 ml/min/1.73 m^2^.

When we compared eGFR by SHID with OS equation, there was a significant difference in GFR ≥90 ml (p-value 0.04), while there was no difference in other categories (Table-II).

Table-III shows comparison of eGFR in children aged ≤ 2 years (n=62) and in above two years (n=16). In children ≤ 2 years, there was significant difference in eGFR by US with OS (p=0.028) in category of ≥90 ml/min /1.73 m^2^ but there was no difference in other GFR categories. While comparing SHID with OS, there was no significant difference in any of eGFR categories. In children above two years, there was no significant difference on comparison of eGFR by US and SHID formula with OS in any eGFR category.

**Table-III:** Comparison of eGFR in Children less than or 2 years and above 2 years (N=78).

	*Original Schwartz formula*	*Updated Schwartz*	*Simple height independent*	US** vs OS*	SHID*** vs OS
***Age ≤ 2yrs(n=62)***					
GFR Mean ± SD	67.97±48.38	55.56±32.93	57.74±31.92		
≥90ml N (%)	15(24.19)	5(8.06)	8(12.9)	0.028	0.165
60-89 ml	14(22.58)	18(29.03)	17(27.42)	0.538	0.678
30-59 ml	22(35.48)	24(87.1)	24(87.1)	0.852	0.852
15-29 ml	7(11.29)	11(17.74)	10(16.13)	0.444	0.601
<15 ml	4(6.45)	4(6.45)	3(4.84)	0.100	0.697
***Age >2 yrs(n=16)***					
GFR Mean ± SD	84.96±54.86	67.77±39.70	65.49±37.14		
≥90 ml	6(37.5)	4(25)	2(12.5)	0.445	0.102
60-89 ml	4(25)	4(25)	5(31.25)	>0.999	0.694
30-59 ml	5(31.25)	7(43.75)	8(50)	0.465	0.281
15-29 ml	1(6.25)	0	1(6.25)	0.500	>0.999
<15 ml	0	1(6.25)	0	0.500	---

* OS: Original Schwartz formula,

** US: updated Schwartz,

*** SHID: Simple height independent

## DISCUSSION

Determination of GFR accurately and its monitoring is important tool in diagnosing and management of acute and CKD in early stages to avoid end stage renal failure.^1^-^3^ Serum creatinine is commonly used biomarker of renal function in clinical practice. Though, it is easily measured and available but it levels depends upon muscle mass which varies greatly with age, height, protein intake, and nutritional status.^4^,^7^ Schwartz formula is most commonly used, though many equations have been developed after Schwartz like US, SHID but OS is still accepted as reliable for estimating GFR in various stage of kidney damage.^1^,^5^,^7^

This study is unique since it was conducted in severely malnourished children below 60 months who were hospitalized due to one or more than one complications of SAM like diarrhea and pneumonia. Since, subnormal GFR was observed in most of study population (73%) and among children with subnormal GFR, majority (47.36%) were falling in GFR between 30-59 ml/min/1.73.^2^ This could be explained based on various complications with which majority of children with SAM were admitted. These findings have been published recently by us.^12^ Significant proportion of children (79.48%) were ≤ 2 years so this may also be explained on the maturity of kidney function as we know that GFR become equal to adult level by the age of 18-24 months.^7^

The clinical application of Schwartz formula has been validated in many studies using either iohexol or Cystatin-C or inulin based clearance.^6^,^13^-^15^ Stanely et al. compared the Schwartz with measured Ccr and found similar results of GFR estimation and recommended that Schwartz formula can be used for routine screening children for CKD.^4^

In present study, our main objective was to compare the eGFR by US and SHID formula with OS and we found that there was a significant difference (0.001) in overall mean eGFR by these formulae as well as in eGFR levels ≥ 90 ml /min/1.73 m^2^, in all age groups but no difference in eGFR categories below 90 ml by either formula. This may suggest that in addition to OS formula the US and SHID may be considered as useful in estimating GFR in all cases of mild to severe renal impairment (eGFR< 90 ml/min/1.73 m^2^).

When we compared eGFR in children ≤ 2 years, there was significant difference in eGFR by US with OS (p=0.028) in category of ≥90 ml/min /1.73 m^2^ but there was no difference in other GFR categories (< 90ml/min/1.73 m^2^) and no difference with comparison of other formulas in any GFR category. So, in children ≤ 2 years; all three formulas were comparable in estimating GFR <90ml categories in our study. Similar variation in eGFR has been found in other studies.^16^-^18^

In children above two years, there was no significant difference on comparison of US and SHID formula with OS in any eGFR category. So, in children above two years US and SHID formulas were comparable with OS.

Though we know that S. Cr is dependent on body mass and in our study children were severely malnourished and we expect low S. Cr and thus higher eGFR as shown by Hari P et al but we could not observe this over estimation of GFR and majority of children in our study had eGFR< 90 ml/min/1.73m^2^.^10^,^19^-^21^ This failure of finding the expected correlation can be explained on the basis that our children with SAM were admitted with one or more than one complications which may have affected the serum creatinine and eGFR.

Hari P et al. has found in regression analysis of local data that value of K as 0.42 in Indian children where S. Cr was measured by Jaffe method, same method was used in the current study.^8^ Consistent with our findings of variation in eGFR above 90 ml/min/1.73 m^2^, Bacchatta et al and Pottel et al have shown that US formula are not accurate above GFR 90 ml/min/1.73m^2^.^22^,^23^

### Limitations

We did not compare US and SHID formula with standard inulin clearance because all children were admitted due to complications of malnutrition and had has no primary renal disease.

## CONCLUSION

We found a significant difference in eGFR in ranges above 90 ml/min/1.73 m^2^ by Updated Schwartz and Original Schwartz in children below two years compared to children above two years. However, there was no difference in GFR categories below 90 ml/min /1.73 m^2^ calculated by either of formula in both age groups. So, it is concluded that either of formula can be used in clinical practice for eGFR in mild to severe renal dysfunction in severely malnourished children. However, more evidence is needed in children with SAM and chronic kidney disease.

### Author’s Contribution

***MA:*** Conception, Data computing, interpretation and draft writing and reviewing final format.

***KNM:*** Conceptualized the idea, interpretation, critical review, write up and final approval.

***BN & SK:*** Data Collection, interpretation, literature review and editing of the manuscript.

***AHM:*** Data analysis.
